# IL-10 produces a dual effect on OGD-induced neuronal apoptosis of cultured cortical neurons via the NF-κB pathway

**DOI:** 10.18632/aging.102411

**Published:** 2019-12-04

**Authors:** Hongbin Chen, Wei Lin, Peiqiang Lin, Mouwei Zheng, Yongxing Lai, Manli Chen, Yixian Zhang, Jianhao Chen, Xiaohui Lin, Longzai Lin, Quan Lan, Qilin Yuan, Ronghua Chen, Xinhong Jiang, Yingchun Xiao, Nan Liu

**Affiliations:** 1 Department of Neurology, Fujian Medical University Union Hospital, Fuzhou, China; 2 Department of Rehabilitation, Fujian Medical University Union Hospital, Fuzhou, China; 3 Institute of Cerebral Vascular Diseases of Fujian Province, Fuzhou, China; 4 Key Laboratory of Brain Aging and Neurodegenerative Diseases, Fujian Key Laboratory of Molecular Neurology, Fujian Medical University, Fuzhou, China; 5 Department of Neurology, The First Affiliated Hospital of Xiamen University, Xiamen, China

**Keywords:** IL-10, primary cortical neurons, OGD, neuronal apoptosis, NF-κB

## Abstract

As a classic immunoregulatory cytokine, interleukin-10 (IL-10) can provide in vivo and in vitro neuroprotection respectively during cerebral ischemia and after the oxygen-glucose deprivation (OGD)-induced injury. However, its role in cortical neuronal survival at different post-ischemic phases remains unclear. The current study found that IL-10 had distinct effects on the neuronal apoptosis at different OGD stages: at an early stage after OGD, IL-10 promoted the OGD-induced neuronal apoptosis in the cultured primary cortical neurons by activating p65 subunit, which up-regulated Bax expression and down-regulated Bcl-xL expression; at a late OGD stage, however, it attenuated the OGD-induced neuronal apoptosis by activating c-Rel, which up-regulated Bcl-xL expression and down-regulated Bax expression. The early-stage pro-apoptosis and late-stage anti-apoptosis were both partly abolished by PDTC, an NF-κB inhibitor, and promoted by PMA, an NF-κB activator. The optimal anti-apoptotic effect appeared when the cultured neurons were treated with IL-10 at 9-24 h after OGD. Taken together, our findings suggest that IL-10 exerts a dual effect on the survival of the cultured neurons by activating the NF-κB pathway at different stages after OGD injury and that PMA treatment at a late stage can facilitate the IL-10-conferred neuroprotection against OGD-induced neuronal injury.

## INTRODUCTION

As a major cause of mortality and disability worldwide, stroke often ensues from impaired blood supply to the brain [1]. After cerebral ischemia, the subsequent inflammatory activation and release of inflammatory cytokines can result in neuronal apoptosis, which ultimately induces severe neurologic deterioration [1–3].

As a prototypical anti-inflammatory cytokine, interleukin-10 (IL-10) can suppress pro-inflammatory signals and immune responses [4, 5]. It has been demonstrated to attenuate the neuronal damage after cerebral infarction [4–6]. Studies with IL-10-deficient mice have also reported a larger infarct volume and higher pro-inflammatory cytokine release after permanent middle cerebral artery occlusion (pMCAO) [4, 7]. This anti-inflammatory effect has also been observed in the molecular line of research. In vitro studies have found that the treatment with IL-10 can produce a concentration-dependent protection for the cultured primary cortical neurons that are exposed to oxygen-glucose deprivation (OGD), in which the neuronal apoptosis can be reduced by up-regulating Bcl-2 expression and down-regulating Bax expression [8, 9]. However, it remains sparsely understood at which post-ischemic stage IL-10 produces its neuronal protective effect.

In neurons, IL-10 can activate the nuclear factor kappa B (NF-κB) [10, 11]. The latter, as a ubiquitous transcriptional factor, has been documented to be involved in the processes of inflammation and apoptosis and can be activated during the development of cerebral ischemia [12], but findings regarding its role in ischemic injury are complex and conflicting. Some studies have reported that NF-κB can promote cell apoptosis by up-regulating the expression of pro-apoptotic factors including Bax [13–16]; whereas, others report it as a survival factor for neurons in cerebral ischemia by up-regulating the Bcl-xL expression and down-regulating the Bax expression in neurons [10, 11, 17–19]. Still more, NF-κB has been reported to perform a dual role in cerebral ischemia, which shows that hypoxia-ischemia induced two peaks of cerebral NF-κB activity: an early NF-κB activation contributing to neuronal damage by down-regulating Bcl-2 and Bcl-xL expression and a late NF-κB activation producing a neuronal protection by increasing Bcl-2 and Bcl-xL expression [12]. But it remains unclear why NF-κB has a dual effect on the brain damage at different post-ischemic stages.

In fact, NF-κB is composed of five subunits of the Rel family, including p65 (RelA), c-Rel, RelB, NF-κB1 (p50), and NF-κB2 (p52). Among these five subunits, p65 and c-Rel are closely related to the neuronal survival [20, 21] and act as opposite regulators of neuronal vulnerability to ischemia. The activation of p65 contributes to the ischemic neuronal damage, while activated c-Rel promotes neuronal resistance to hypoxia [21]. In vivo studies have documented that p65 is mainly activated in neurons at an early stage after the middle cerebral artery occlusion (MCAO), which contributes to the post-ischemic neurodegenerative process [22]. This finding has been confirmed by in vitro studies, which evidence that p65, but not c-Rel, is highly activated in neurons 3 hours after OGD [21]. Therefore, the mediation of the expression of p65 subunit and c-Rel subunit may be involved in the observed dual effect of NF-κB on cerebral ischemia.

Taken together, given the findings and facts: the activation of NF-κB by IL-10 and the dual role of NF-κB activation in neuronal damage after hypoxia-ischemia, it would be intriguing and rewarding to speculate whether IL-10 can produce a dual effect on OGD-injured cultured cortical neurons at different post-ischemic stages and whether p65 subunit and c-Rel subunit are involved in its hypothesized effects.

In the present work, we exposed cortical neurons to OGD injury to build a cerebral ischemic model in vitro and treated them with IL-10 and an NF-κB inhibitor (PDTC) and an activator (PMA) at different stages. We found that IL-10 produced a dual effect on the apoptosis of cortical neurons at different OGD stages by mediating the p65 pathway at an early stage and c-Rel pathway at a late stage, and that IL-10 administration showed an optimal neuronal protective effect at a middle-to-late stage after OGD. These findings provide new insight into the role of IL-10 in cerebral ischemia and its potential therapeutic value.

## RESULTS

### OGD induces peaks of p65 and c-Rel in the cultured primary cortical neurons at different OGD stages

**Figure 1:**
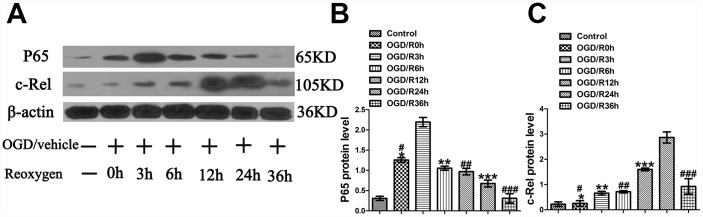
**OGD-induced peaks of p65 and c-Rel at different time points in cultured primary cortical neurons after OGD injury.** (**A**) The representative image of western blot analysis for p65 and c-Rel. (**B**) Western blot analysis of p65 (n=3). *p<0.001, as compared with Control group; ^#^*p*<0.001, as compared with OGD/R3h group; **p<0.001, as compared with OGD/R3h group; ^##^*p*<0.001, as compared with OGD/R3h group; ***p<0.001, as compared with OGD/R3h group; ^###^*p*<0.001, as compared with OGD/R3h group; by one way analysis of variance (ANOVA) followed by Student-Newman-Keuls multiple comparisons test, F=61.131, *p*<0.0001. (**C**) Western blot analysis of c-Rel (n=3). *p>0.05, as compared with Control group; ^#^*p*<0.001, as compared with OGD/R24h group; **p<0.001, as compared with OGD/R24h group; ^##^*p*<0.001, as compared with OGD/R24h group; ***p<0.001, as compared with OGD/R24h group; ^###^*p*<0.001, as compared with OGD/R24h group; by one way analysis of variance (ANOVA) followed by Student-Newman-Keuls multiple comparisons test, F=78.545, *p*<0.0001.

As the literature indicates that hypoxia-ischemia can induce 2 peaks of NF-κB activity at 3 to 6 h and 24 h in the cerebral cortex of P7 rats after unilateral carotid artery occlusion and hypoxia [12] and that p65, rather than c-Rel, is rapidly activated in neurons after MCAO [22] and OGD [21], it is important to determine the levels of p65 and c-Rel at different time points after OGD. To elucidate the changes of p65 and c-Rel after OGD injury, their levels were measured at 0 h, 3 h, 6 h, 12 h, 24 h and 36 h after OGD. The experimental groups were designed as follows: Control group, OGD/R0h group, and OGD/R3h group, OGD/R6h group, OGD/R12h group, OGD/R24h group, OGD/R36h. The analyses showed that compared with the Control group, the OGD/R0h group reported a higher expression of p65 (1.26±0.06 vs. 0.31±0.05, *p*<0.001) (Figure 1A, 1B); compared with the OGD/R0h group, OGD/R6h group, OGD/R12h group, OGD/R24h group, and OGD/R36h group, the OGD/R3h group reported a higher expression of p65 (2.20±0.11 vs. 1.26±0.06, 2.20±0.11 vs. 1.05±0.05, 2.20±0.11 vs. 0.97±0.08, 2.20±0.11 vs*.*0.68±0.09, 2.20±0.11 vs. 0.31±0.12, *p*<0.001, respectively) (Figure 1A, 1B). Meanwhile, western blotting revealed no significant difference in c-Rel expression between the Control group and the OGD group (0.22±0.09 vs. 0.25±0.11, *p>*0.05) (Figure 1A, 1C). In comparison with the OGD/R0h group, OGD/R3h group, OGD/R6h group, OGD/R12h group, and OGD/R36h group, the OGD/R24h group showed a higher c-Rel expression (2.87±0.13 vs. 0.25±0.11, 2.87±0.13 vs. 0.66±0.07, 2.87±0.13 vs. 0.71±0.04, 2.87±0.13 vs. 1.60±0.05, 2.87±0.13 vs. 0.93±0.18, *p*<0.001, respectively) (Figure 1A, 1C). These results showed that the expression peak of p65 appeared at 3 h after OGD injury and that of c-Rel activity did at 24 h after OGD injury.

### The effect of IL-10 on OGD-induced apoptosis in cultured primary cortical neurons at different time points after OGD injury

**Figure 2:**
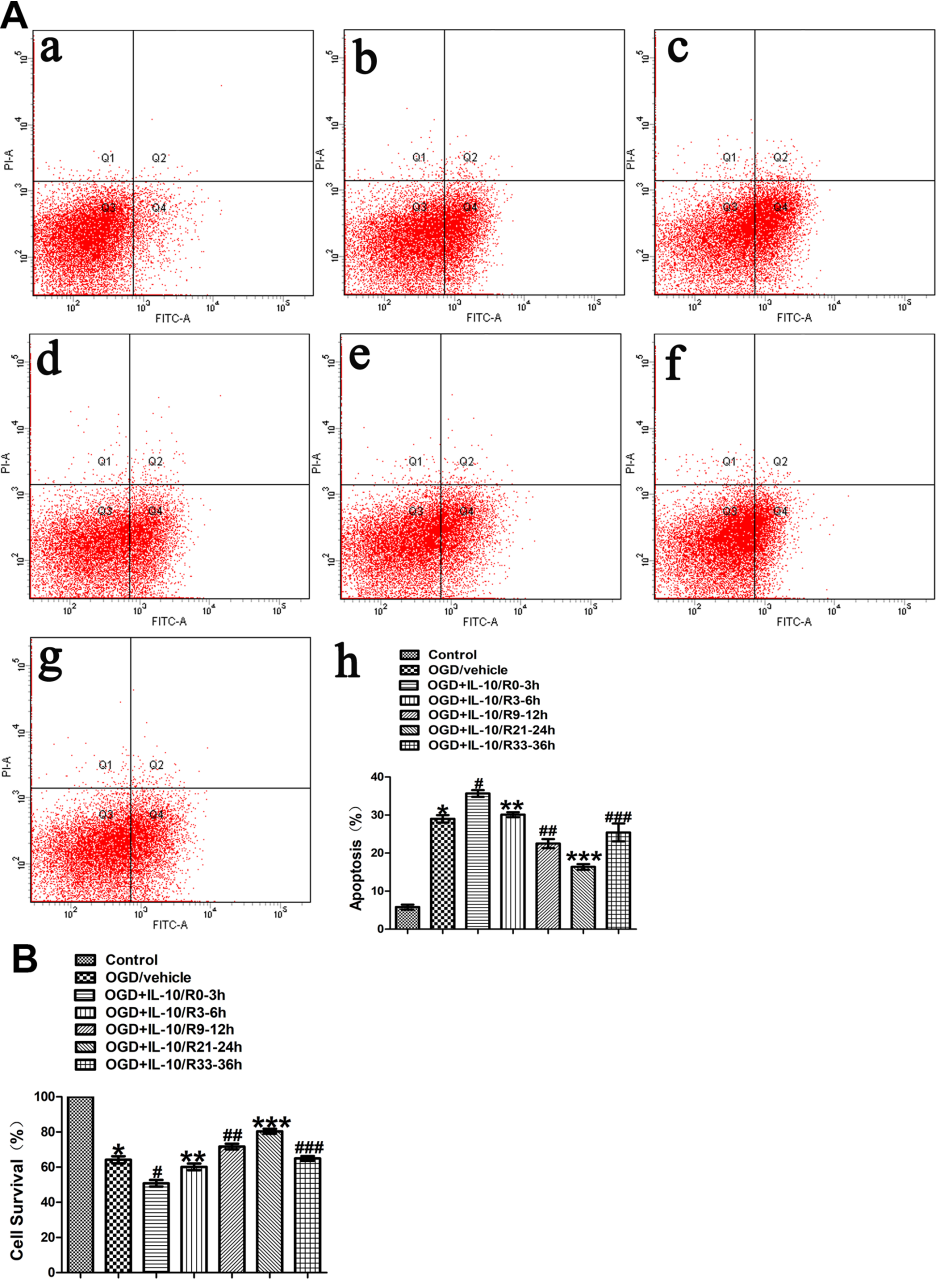
**The effect of IL-10 on OGD-induced apoptosis in cultured primary cortical neurons at different time points after OGD injury.** (**A**) Forty-eight hours after OGD, the apoptosis of neurons was detected by flow cytometry. The signals from apoptotic neurons were localized in the Q2 and Q4 quadrants of the resulted dot-plot graph. (a) Control group; (b) OGD group; (c) OGD+IL-10/R0-3h group; (d) OGD+IL-10/R3-6h; (e) OGD+IL-10/R9-12h; (f) OGD+IL-10/R21-24h; (g) OGD+IL-10/R33-36h; (h) Statistical graph of apoptosis in different groups (n=3). *p<0.001, as compared with Control group; ^#^*p*<0.01, as compared with OGD group; **p>0.05, as compared with OGD group; ^##^*p*<0.01, as compared with OGD group; ***p<0.001, as compared with OGD group; ^###^*p*>0.05, as compared with OGD group; by one way analysis of variance (ANOVA) followed by Student-Newman-Keuls multiple comparison test, F=69.591, *p*<0.0001. (**B**) Forty-eight hours after OGD, survival of neurons was detected by MTT. Statistical graph of cell survival in different groups (n=3). *p<0.001, as compared with Control group; ^#^*p*<0.001, as compared with OGD group; **p>0.05, as compared with OGD group; ^##^*p*<0.05, as compared with OGD group; ***p<0.001, as compared with OGD group; ^###^*p*>0.05, as compared with OGD group; by one way analysis of variance (ANOVA) followed by Student-Newman-Keuls multiple comparison test, F=102.550, *p*<0.0001.

To evaluate the effect of IL-10 on neuronal apoptosis at different time points after OGD injury, neurons were treated with IL-10 at 0-3 h, 3-6 h, 9-12 h, 21-24 h and 33-36 h after OGD, respectively. The experimental groups were designed as follows: Control group, OGD group, and OGD+IL-10/R0-3h group, OGD+IL-10/R3-6h group, OGD+IL-10/R9-12h group, OGD+IL-10/R21-24h group, OGD+IL-10/R33-36h. Flow cytometry assay showed that the OGD treatment significantly increased the apoptosis rate when the OGD group was compared with the Control group (29.00±0.97% vs. 5.80±0.64%, *p*<0.001) (Figure 2A). Compared with the OGD group, the OGD+IL-10/R0-3h group reported a higher apoptosis rate (35.67±0.87% vs. 29.00±0.97%, *p*<0.01); the OGD+IL-10/R9-12h group and OGD+IL-10/R21-24h group showed a significantly lower apoptosis rate (22.50±1.17% vs. 29.00±0.97%, *p*<0.01; 16.37±0.73% vs. 29.00±0.97%, *p*<0.001, respectively) (Figure 2A); no significant difference was found between the OGD group and the OGD+IL-10/R3-6h group or OGD+IL-10/R33-36h group (29.00±0.97% vs. 30.10±0.65%, *p*>0.05; 29.00±0.97% vs. 25.43±2.34%, *p*>0.05, respectively) (Figure 2A). These results demonstrate that IL-10 treatment at 0-3 h after OGD results in apoptosis promotion in OGD-injured neurons and that IL-10 administration at 9-12 h and 21-24 h after OGD can significantly suppress OGD-induced neuronal apoptosis.

MTT was further performed to measure neuronal viability of each group. The assay showed that the OGD treatment significantly decreased the cortical neuronal viability when the OGD group was compared with the Control group (64.18±2.07% vs. 100%, *p*<0.001) (Figure 2B). When different OGD time points were considered, the cell viability varied when different groups were compared with the OGD group: the OGD+IL-10/R0-3h group reported a significantly lower cell viability (50.80±1.88% vs. 64.18±2.07%, *p*<0.001) and the OGD+IL-10/R3-6h group showed no significant difference (60.16±1.89% vs. 64.18±2.07%, *p*>0.05) (Figure 2B); the OGD+IL-10/R9-12h group and OGD+IL-10/R21-24h group both evidenced a higher neuronal viability (respectively, 71.71±1.59% vs. 64.18±2.07%, *p*<0.05; 80.41±1.34% vs. 64.18±2.07%, *p*<0.001) and the OGD+IL-10/R33-36h group reported no significant difference (64.18±2.07% vs. 64.97±1.34%, *p*>0.05) (Figure 2B). The peak and nadir apoptotic rates were respectively observed at 0-3 h and 21-24 h after OGD injury. These results confirm that the IL-10 treatment can induce a marked cell viability reduction in the neurons at 0–3 h after OGD and promote the neuronal viability when it is added to OGD-injured neurons at 9–12 h and 21–24 h after OGD injury, suggesting that IL-10 can produce a pro-apoptotic at an early stage and an anti-apoptotic effect at a mid-to-late stage on the OGD-injured neurons.

### IL-10 increases OGD-induced apoptosis in cultured primary cortical neurons via p65 at an early OGD stage

Further experiments were conducted to elucidate the mechanism of IL-10-induced early pro-apoptotic effect on neurons after OGD injury. The neurons were simultaneously treated with IL-10 and PDTC (a specific inhibitor of NF-κB) or PMA (an activator of NF-κB) immediately after OGD to further investigate whether p65 pathway is involved in the early pro-apoptotic effect of IL-10. Three hours after drug treatment, the medium was changed to a neuronal medium which contained no IL-10, PDTC and PMA. The groups were designated as follows: Control group, OGD group, OGD+IL-10/R0-3h group and OGD+IL-10/R0-3h+PDTC/R0-3h group, OGD+IL-10/R0-3h+PMA/R0-3h group. After three hours of drug treatment, western blotting was employed to evaluate the protein expression of p65. Forty-eight hours after OGD, flow cytometry assay and western blotting were performed to evaluate apoptosis.

**Figure 3:**
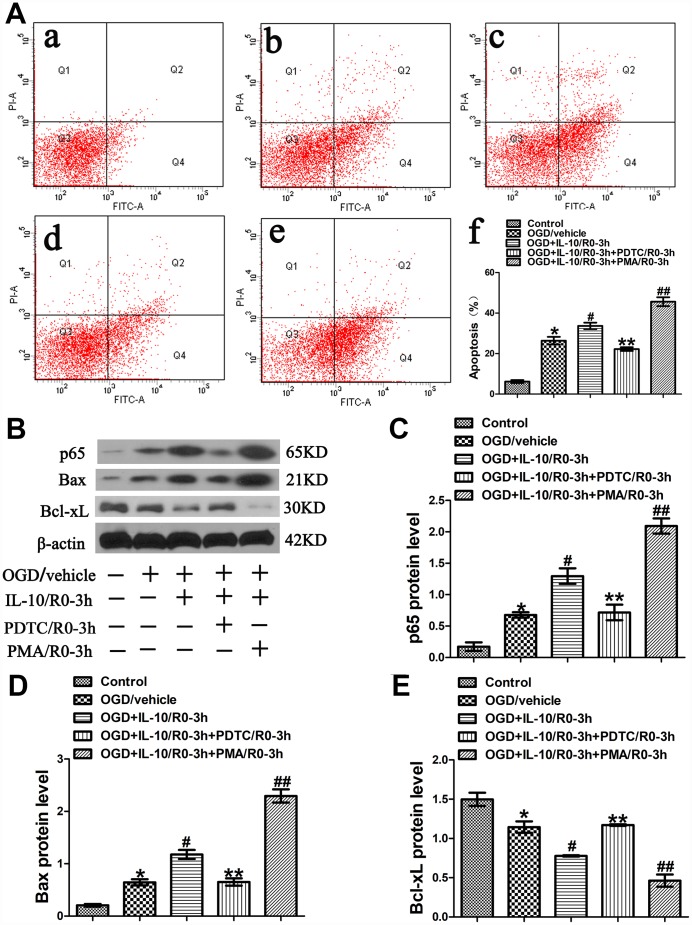
**OGD-induced apoptosis increased by IL-10 via p65 at an early stage after OGD injury.** (**A**) Forty-eight hours after OGD, apoptosis of neurons was detected by flow cytometry. The signals from apoptotic neurons were localized in the Q2 and Q4 quadrants of the resulting dot-plot graph. (a) Control group; (b) OGD group; (c) OGD+IL-10/R0-3h group; (d) OGD+IL-10/R0-3h+PDTC/R0-3h; (e) OGD+IL-10/R0-3h+PMA/R0-3h group; (f) Statistical graph of apoptosis in different groups (n=3). *p<0.001, as compared with Control group; ^#^*p*<0.01, as compared with OGD group; **p<0.01, as compared with OGD+IL-10/R0-3h group; ^##^*p*<0.001, as compared with OGD+IL-10/R0-3h group; by one way analysis of variance (ANOVA) followed by Student-Newman-Keuls multiple comparison test, F=85.931, *p*<0.0001. (**B**) The representative image of western blot analysis for p65, Bax and Bcl-xL expression. (**C**) Western blot analysis of p65 (n=3). *p<0.01, as compared with Control group; ^#^*p*<0.01, as compared with OGD group; **p<0.01, as compared with OGD+IL-10/R0-3h group; ^##^*p*<0.001, as compared with OGD+IL-10/R0-3h group; by one way analysis of variance (ANOVA) followed by Student-Newman-Keuls multiple comparison test, F=52.058, *p*<0.0001. (**D**) Western blot analysis of Bax (n=3). *p<0.01, as compared with Control group; ^#^*p*<0.01, as compared with OGD group; **p<0.01, as compared with OGD+IL-10/R0-3h group; ^##^*p*<0.001, as compared with OGD+IL-10/R0-3h group; by one way analysis of variance (ANOVA) followed by Student-Newman-Keuls multiple comparison test, F=100.199, *p*<0.0001. (**E**) Western blot analysis of Bcl-xL (n=3). *p<0.01, as compared with Control group; ^#^*p*<0.01, as compared with OGD group; **p<0.01, as compared with OGD+IL-10/R0-3h group; ^##^*p*<0.01, as compared with OGD+IL-10/R0-3h group; by one way analysis of variance (ANOVA) followed by Student-Newman-Keuls multiple comparison test, F=43.085, *p*<0.0001. All data are presented as mean±SEM.

Results from flow cytometry assay showed that the OGD treatment significantly increased the neuronal apoptosis when the OGD group was compared with the Control group (26.37±1.91% vs. 6.20±0.71%, *p*<0.001) (Figure 3A). Moreover, as indicated in Figure 3A, compared with the OGD group, the OGD+IL-10/R0-3h group reported a higher apoptosis rate (33.67±1.59% vs. 26.37±1.91%, *p*<0.01); compared with that of the OGD+IL-10/R0-3h group, the apoptosis rate obviously decreased in the OGD+IL-10/R0-3h+PDTC/R0-3h group, but significantly increased in the OGD+IL-10/R0-3h+PMA/0-3h group (22.23±0.91% vs. 33.67±1.59%, *p*<0.01; 45.63±2.19% vs. 33.67±1.59%, *p*<0.001, respectively); no significant difference was found between the OGD group and OGD+IL-10/R0-3h+PDTC/R0-3h group (26.37±1.91% vs. 22.23±0.91%, *p*＞0.05).

To further elucidate the molecular mechanism by which IL-10 exerts a pro-apoptotic effect on neurons at the early stage after OGD injury, western blotting was performed to analyze the expression of p65, Bax and Bcl-xL. The analysis found that the OGD treatment increased p65 expression when the OGD group was compared with the Control group (0.68±0.04 vs. 0.17±0.07, *p*＜0.01) (Figure 3B, 3C); furthermore, compared with the OGD group, the OGD+IL-10/R0-3h group presented a higher protein expression of p65 (1.30±0.12 vs. 0.68±0.04, *p*＜0.01) (Figure 3B, 3C); however, when compared with that of the OGD+IL-10/R0-3h group, the protein expression of p65 was much lowered in the OGD+IL-10/R0-3h+PDTC/R0-3h group, but markedly increased in the OGD+IL-10/R0-3h+PMA/0-3h group (0.72±0.12 vs. 1.30±0.12, *p*＜0.01; 2.09±0.12 vs. 1.30±0.12, *p*＜0.001, respectively) (Figure 3B, 3C).

Western blot results also indicated that neurons exposed to the OGD treatment showed a higher expression of Bax but a lower expression of Bcl-xL when the OGD group was compared with the Control group (Bax, 0.65±0.06 vs. 0.21±0.03, *p*<0.01; Bcl-xL, 1.14±0.07 vs. 1.50±0.08, *p*<0.01) (Figure 3B, 3D, 3E). When compared with the OGD group, the OGD+IL-10/R0-3h group displayed a significant increase in Bax expression and a marked decrease in Bcl-xL expression (Bax, 1.18±0.09 vs. 0.65±0.06, *p*<0.01; Bcl-xL, 0.78±0.01 vs. 1.14±0.07, *p<*0.01, respectively) (Figure 3B, 3D, 3E). Moreover, compared with the OGD+IL-10/R0-3h group, the OGD+IL-10/R0-3h+PDTC/R0-3h group reported a lower Bax expression but a higher Bcl-xL expression (Bax, 0.65±0.07 vs. 1.18±0.09; Bcl-xL, 1.17±0.01 vs. 0.78±0.01; *p*<0.01, respectively) (Figure 3B, 3D, 3E), while the OGD+IL-10/R0-3h+PMA/R0-3h group reported a much higher Bax expression but a lower Bcl-xL expression (Bax, 2.29±0.13 vs. 1.18±0.09, *p*<0.001; Bcl-xL, 0.46±0.08 vs. 0.78±0.01, *p*<0.01) (Figure 3B, 3D, 3E).

These results demonstrate that IL-10 activates p65 expression and accelerates OGD-induced apoptosis in neurons by up-regulating the expression of pro-apoptotic protein Bax and down-regulating the expression of the anti-apoptotic protein Bcl-xL at an early stage after OGD injury. These effects can be partially blocked by NF-κB inhibitor PDTC but facilitated by NF-κB promoter PMA.

### IL-10 enhances p65 nuclear translocation in cultured primary cortical neurons at an early stage after OGD injury

**Figure 4:**
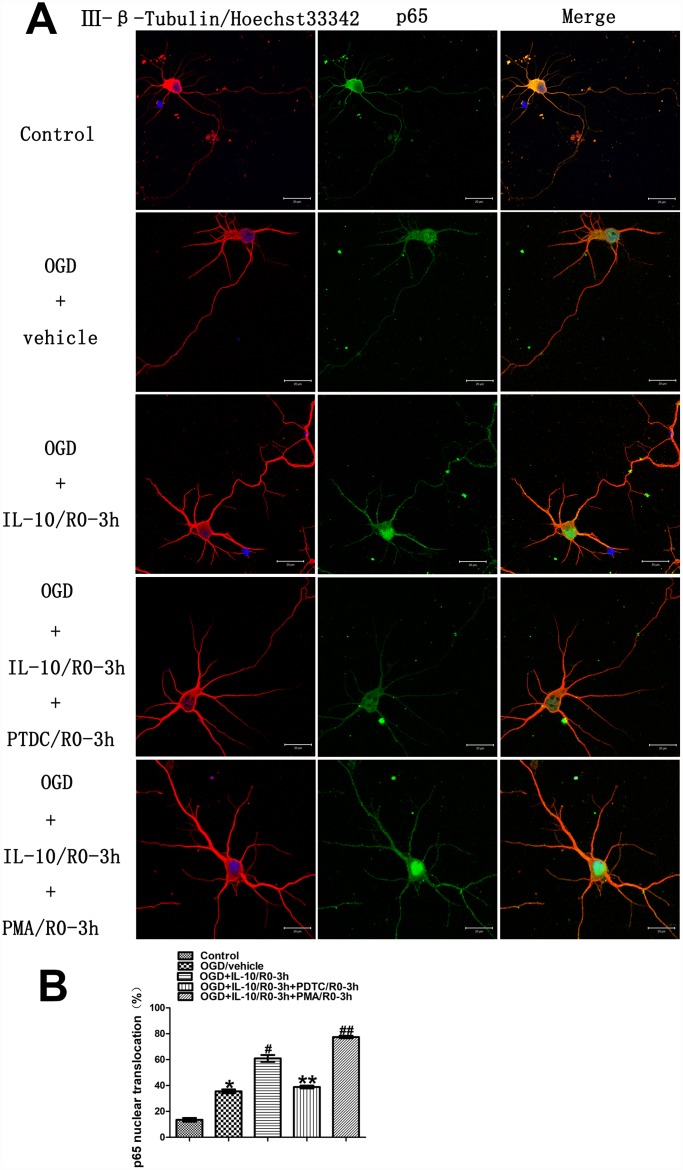
**Localization and nuclear translocation of p65 at an early stage after OGD injury.** (**A**) Representative images of each group. The left column displays neuronal marker class Ⅲ-β-Tubulin (red) and nucleus (blue). The middle column shows expression of p65 (green). The right column indicates the co-localization of class Ⅲ-β-Tubulin, nucleus and P65. Scale bar is 20 μm. (**B**) Quantification of p65 nuclear translocation. *p<0.001, as compared with Control group; ^#^*p*<0.001, as compared with OGD group; **p<0.001, as compared with OGD+IL-10/R0-3h group; ^##^*p*<0.001, as compared with OGD+IL-10/R0-3h group; by one way analysis of variance (ANOVA) followed by Student-Newman-Keuls multiple comparison test, F=225.278, *p*<0.0001. All data are presented as mean±SEM.

In order to determine whether IL-10 enhances p65 nuclear translocation in the cultured primary cortical neurons at an early stage after OGD injury, we examined the localization and quantified the nuclear translocation of p65 by immunofluorescence staining 3 hours after drug treatment. The groups were designed as follows: Control group, OGD group, OGD+IL-10/R0-3h group and OGD+IL-10/R0-3h+PDTC/R0-3h group, OGD+IL-10/R0-3h+PMA/R0-3h group. As shown in Figure 4, in the Control group, the immunoreactivity of p65 was present in the cytoplasm (green fluorescence) but was almost absent from the nucleus. After 2 hours of exposure to OGD, weak green fluorescence was distributed in the nucleus and the nuclear translocation of p65 increased when compared with the Control group (35.56±1.49% vs. 13.56±1.40%, *p*<0.001) (Figure 4A and 4B). Moreover, compared with the OGD group, the IL-10 treatment after OGD injury enhanced the nuclear translocation of p65 in the OGD+IL-10/R0-3h group (60.97±2.67% vs. 35.56±1.49%, *p*<0.001) (Figure 4A and 4B), which indicates the transference of small portions of p65 protein from the cytoplasm to the nucleus. Compared with that of the OGD+IL-10/R0-3h group, the nuclear translocation of p65 decreased in the OGD+IL-10/R0-3h+PDTC/R0-3h group but significantly increased in the OGD+IL-10/R0-3h+PMA/R0-3h group (38.88±1.10% vs. 60.97±2.67%, *p*<0.001; 77.47±0.97% vs. 60.97±2.67%, *p*<0.001, respectively) (Figure 4A and 4B). These changes clearly demonstrate that IL-10 enhances the nuclear translocation of p65 in cultured cortical neurons at an early stage after OGD injury and that this translocation can be blocked by PDTC and activated by PMA.

### IL-10 suppresses OGD-induced apoptosis in cultured primary cortical neurons via c-Rel at a late OGD stage

In order to further explore the mechanism of IL-10-induced anti-apoptotic effect on neurons at a late stage after OGD injury, IL-10, PDTC, and PMA were simultaneously added into OGD-injured neurons at 21 h after OGD to further investigate whether c-Rel pathway is involved in the anti-apoptotic effect of IL-10 at a late stage after OGD injury. Three hours after drug treatment, the medium was changed to a neuronal medium which contained no IL-10, PDTC and PMA. The groups were as follows: Control group, OGD group, OGD+IL-10/R21-24h group and OGD+IL-10/R21-24h+PDTC/R21-24h group, OGD+IL-10/R21-24h+PMA/R21-24h group. After three hours of drug treatment, western blot analysis was adopted to evaluate the protein expression of c-Rel. Forty-eight hours after OGD, flow cytometry assay and western blotting were performed to evaluate the apoptosis of each group.

**Figure 5:**
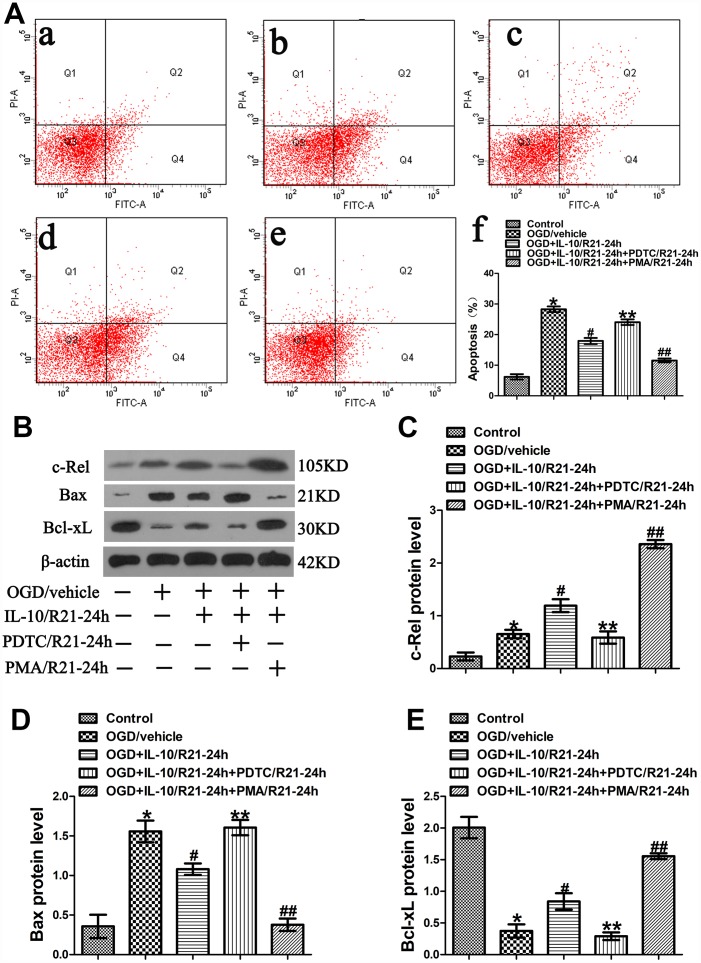
**OGD-induced apoptosis decreased by IL-10 via c-Rel at a late stage after OGD injury.** (**A**) Forty-eight hours after OGD, apoptosis of neurons was detected by flow cytometry. The signals from apoptotic neurons were localized in the Q2 and Q4 quadrants of the resulting dot-plot graph. (a) Control group; (b) OGD group; (c) OGD+IL-10/R21-24h group; (d) OGD+IL-10/R21-24h+PDTC/R21-24h; (e) OGD+IL-10/R21-24h+PMA/R21-24h group; (f) Statistical graph of apoptosis in different groups (n=3). *p<0.001, as compared with Control group; ^#^*p*<0.001, as compared with OGD group; **p<0.001, as compared with OGD+IL-10/R21-24h group; ^##^*p*<0.001, as compared with OGD+IL-10/R21-24h group; by one way analysis of variance (ANOVA) followed by Student-Newman-Keuls multiple comparison test, F=106.928, *p*<0.0001. (**B**) The representative image of western blot analysis for c-Rel, Bax and Bcl-xL expression. (**C**) Western blot analysis of c-Rel (n=3). *p<0.05, as compared with Control group; ^#^*p*<0.01, as compared with OGD group; **p<0.01, as compared with OGD+IL-10/R21-24h group; ^##^*p*<0.001, as compared with OGD+IL-10/R21-24h group; by one way analysis of variance (ANOVA) followed by Student-Newman-Keuls multiple comparison test, F=74.605, *p*<0.0001. (**D**) Western blot analysis of Bax (n=3). *p<0.001, as compared with Control group; ^#^*p*<0.05, as compared with OGD group; **p<0.05, as compared with OGD+IL-10/R21-24h group; ^##^*p*<0.01, as compared with OGD+IL-10/R21-24h group; by one way analysis of variance (ANOVA) followed by Student-Newman-Keuls multiple comparison test, F=30.082, *p*<0.0001. (**E**) Western blot analysis of Bcl-xL (n=3). *p<0.001, as compared with Control group; ^#^*p*<0.05, as compared with OGD group; **p<0.05, as compared with OGD+IL-10/R21-24h group; ^##^*p*<0.01, as compared with OGD+IL-10/R21-24h group; by one way analysis of variance (ANOVA) followed by Student-Newman-Keuls multiple comparison test, F=45.201, *p*<0.0001. All data are presented as mean±SEM.

Results from flow cytometry assay showed that the OGD treatment significantly aggravated the neuronal apoptosis when the OGD group was compared with the Control group (28.30±0.90% vs. 6.20±0.87%, *p*<0.001) (Figure 5A). Moreover, as indicated in Figure 5A, compared with the OGD group, the OGD+IL-10/R21-24h group reported a lower apoptosis rate (17.93±0.97% vs. 28.30±0.90%, *p*<0.001); compared with that of the OGD+IL-10/R21-24h group, the apoptosis rate was higher in the OGD+IL-10/R21-24h+PDTC/R21-24h group, but decreased in the OGD+IL-10/R21-24h+PMA/21-24h group (24.07±0.92% vs. 17.93±0.97%, *p*<0.001; 11.57±0.64% vs. 17.93± 0.97%, *p*<0.001, respectively); no significant difference was found between the OGD group and OGD+IL-10/R21-24h+PDTC/R21-24h group (28.30±0.90% vs. 24.07±0.92%, *p*＞0.05).

To further explore the molecular mechanism by which IL-10 exerts an anti-apoptotic effect on the neurons at a late stage after OGD injury, we analyzed the expression of c-Rel, Bax and Bcl-xL by western blotting. We found that the OGD treatment increased c-Rel expression when the OGD group was compared with the Control group (0.65±0.08 vs. 0.23±0.07, *p*<0.05) (Figure 5B, 5C); furthermore, when compared with the OGD group, the OGD+IL-10/R21-24h group presented a higher protein expression of c-Rel (1.19±0.12 vs. 0.65±0.08, *p*<0.01) (Figure 5B, 5C); however, when compared with that of the OGD+IL-10/R0-3h group, the protein expression of c-Rel was much lowered in the OGD+IL-10/R21-24h+PDTC/R21-24h group, but markedly increased in the OGD+IL-10/R21-24h+PMA/R21-24h group (0.59±0.12 vs. 1.19±0.12, *p*<0.01; 2.36±0.08 vs. 1.19±0.12, *p*<0.001, respectively) (Figure 5B, 5C).

Western blot results indicated that neurons exposed to the OGD treatment showed a higher expression of Bax, but a lower expression of Bcl-xL when the OGD group was compared with the Control group (Bax, 1.56±0.14 vs. 0.36±0.15, *p*<0.001; Bcl-xL, 0.37±0.10 vs. 2.01±0.17, *p*<0.001) (Figure 5B, 5D, 5E). When compared with the OGD group, the OGD+IL-10/R21-24h group displayed a significant decrease in Bax expression and a marked increase in Bcl-xL expression (Bax, 1.08±0.07 vs. 1.56±0.14, *p*<0.05; Bcl-xL, 0.84±0.13 vs. 0.37±0.10, *p*<0.05, respectively) (Figure 5B, 5D, 5E). Moreover, compared with the OGD+IL-10/R21-24h group, the OGD+IL-10/R21-24h+PDTC/ R21-24h group reported a higher Bax expression but a lower Bcl-xL expression (Bax, 1.61±0.10 vs. 1.08±0.07; Bcl-xL, 0.29±0.06 vs. 0.84±0.13; *p*<0.05, respectively) (Figure 5B, 5D, 5E), while the OGD+IL-10/R21-24h+PMA/21-24h group reported a lower Bax expression but a higher Bcl-xL expression (Bax, 0.38±0.08 vs. 1.08±0.07, *p*<0.01; Bcl-xL, 1.56±0.05 vs. 0.84±0.13, *p*<0.01) (Figure 5B, 5D, 5E).

These results show that IL-10 activates c-Rel expression and suppresses OGD-induced apoptosis in neurons by down-regulating the protein level of Bax and up-regulating the protein level of Bcl-xL at a late stage after OGD injury. These effects can be partially blocked by PDTC but facilitated by PMA.

### IL-10 enhances c-Rel nuclear translocation in cultured primary cortical neurons at a late OGD stage

**Figure 6:**
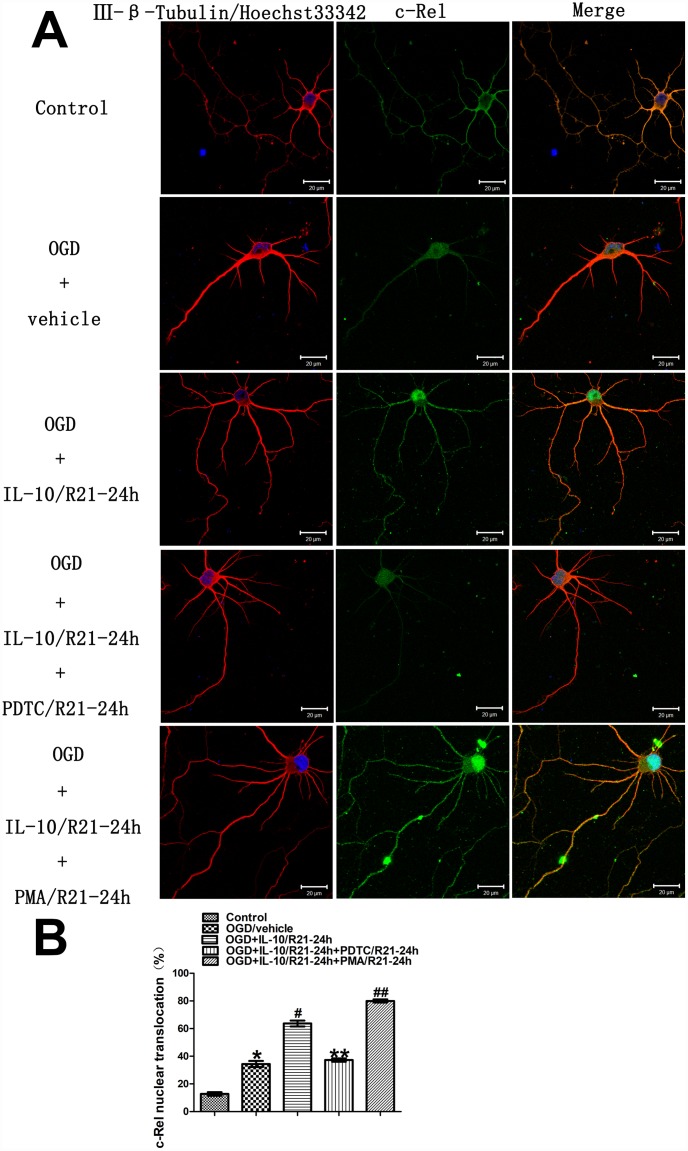
**Localization and nuclear translocation of c-Rel at a late stage after OGD injury.** (**A**) Representative images of each group. The left column displays neuronal marker class Ⅲ-β-Tubulin (red) and nucleus (blue). The middle column shows expression of c-Rel (green). The right column indicates the co-localization of class Ⅲ-β-Tubulin, nucleus and c-Rel. Scale bar is 20 μm. (**B**) Quantification of c-Rel nuclear translocation. *p<0.001, as compared with Control group; ^#^*p*<0.001, as compared with OGD group; **p<0.001, as compared with OGD+IL-10/R21-24h group; ^##^*p*<0.001, as compared with OGD+IL-10/R21-24h group; by one way analysis of variance (ANOVA) followed by Student-Newman-Keuls multiple comparison test, F=254.238, *p*<0.0001. All data are presented as mean±SEM.

In order to ascertain whether IL-10 enhances c-Rel nuclear translocation in the cultured primary cortical neurons at a late OGD stage, immunofluorescence staining was performed to examine the localization and quantify the nuclear translocation of c-Rel at 24 h after OGD. The groups were as follows: Control group, OGD group, OGD+IL-10/R21-24h group and OGD+IL-10/R21-24h+PDTC/R21-24h group, OGD+IL-10/R21-24h+PMA/R21-24h group. As shown in Figure 6, under control condition, the immunoreactivity of c-Rel was visible in the cytoplasm (green fluorescence), but was almost absent from the nucleus. After 2 hours of exposure to OGD, a weak green fluorescence appeared in the nucleus. The nuclear translocation of c-Rel increased when compared with the Control group (34.41±2.19% vs. 12.75±1.29%, *p*<0.001) (Figure 6A and 6B). Furthermore, compared with the OGD group, IL-10 treatment after OGD enhanced the nuclear translocation of c-Rel (63.68±2.11% vs. 34.41±2.19%, *p*<0.001) (Figure 6A and 6B), which indicates a partial transference of c-Rel protein from the cytoplasm to the nucleus. Compared with that of the OGD+IL-10/R21-24h group, the nuclear translocation of c-Rel decreased in the OGD+IL-10/R21-24h+PDTC/R21-24h group but significantly increased in the OGD+IL-10/R21-24h+ PMA/R21-24h group (37.26±1.23% vs. 63.68±2.11%, *p*<0.001; 79.99±1.13% vs. 63.68±2.11%, *p*<0.001, respectively) (Figure 6A and 6B). These changes clearly demonstrate that IL-10 enhances the nuclear translocation of c-Rel in cultured cortical neurons at a late stage after OGD injury and that nuclear translocation of c-Rel can be restrained by PDTC but promoted by PMA.

### The strongest anti-apoptotic effect of IL-10 observed at 9-24 h after OGD

**Figure 7:**
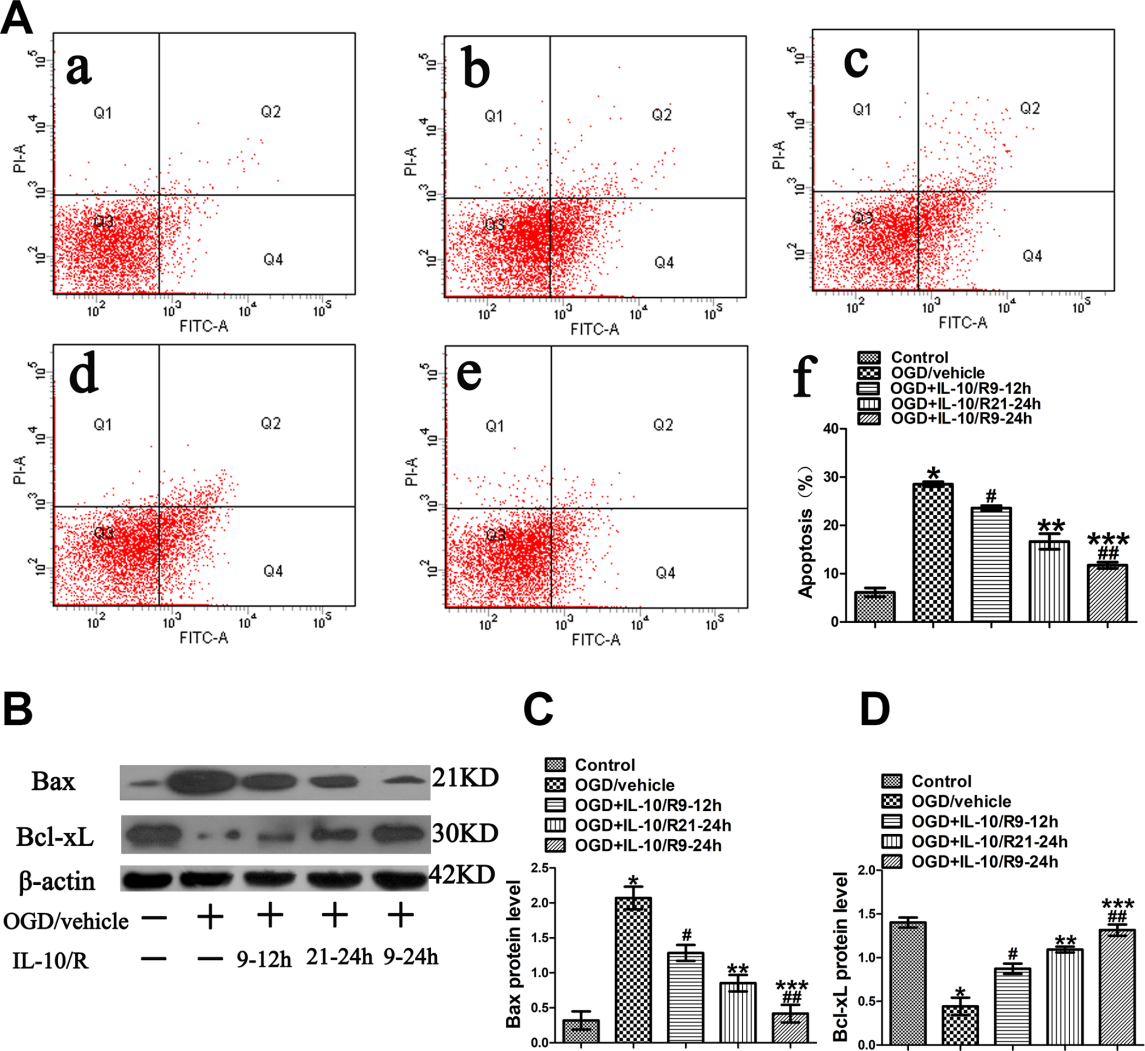
**The strongest anti-apoptotic effect of IL-10 on cultured primary cortical neurons after OGD injury.** (**A**) Forty-eight hours after OGD, apoptosis of neurons was detected by flow cytometry. The signals from apoptotic neurons were localized in the Q2 and Q4 quadrants of the resulting dot-plot graph. (a) Control group; (b) OGD group; (c) OGD+IL-10/R9-12h group; (d) OGD+IL-10/R21-24h; (e) OGD+IL-10/R9-24h; (f) Statistical graph of apoptosis in different groups (n=3). *p<0.001, as compared with Control group; ^#^*p*<0.01, as compared with OGD group; **p<0.001, as compared with OGD group; ^##^*p*<0.001, as compared with OGD+IL-10/R9-12h group; ***p<0.01, as compared with OGD+IL-10/R21-24h group; by one way analysis of variance (ANOVA) followed by Student-Newman-Keuls multiple comparison test, F=92.017, *p*<0.0001. (**B**) The representative image of western blot analysis for Bax and Bcl-xL expression. (**C**) Western blot analysis of Bax (n=3). *p<0.001, as compared with Control group; ^#^*p*<0.01, as compared with OGD group; **p<0.001, as compared with OGD group; ^##^*p*<0.01, as compared with OGD+IL-10/R9-12h group; ***p<0.05, as compared with OGD+IL-10/R21-24h group; by one way analysis of variance (ANOVA) followed by Student-Newman-Keuls multiple comparison test, F=29.782, *p*<0.0001. (**D**) Western blot analysis of Bcl-xL (n=3). *p<0.001, as compared with Control group; ^#^*p*<0.001, as compared with OGD group; **p<0.001, as compared with OGD group; ^##^*p*<0.01, as compared with OGD+IL-10/R9-12h group; ***p<0.05, as compared with OGD+IL-10/R21-24h group; by one way analysis of variance (ANOVA) followed by Student-Newman-Keuls multiple comparison test, F=33.906, *p*<0.0001. All data are presented as mean±SEM.

As shown in Figure 1, IL-10 suppressed OGD-induced apoptosis and promoted neuronal viability if administered to OGD-injured neurons at 9-12 h and 21-24 h after OGD. Therefore, we speculated whether IL-10 would produce the strongest anti-apoptotic effect if added into OGD-injured neurons at 9-24h. The experimental groups were designed as follows: Control group, OGD group, and OGD+IL-10/R9-12h group, OGD+IL-10/R21-24h group, OGD+IL-10/R9-24h group. Results from flow cytometry assay showed that the OGD treatment significantly increased the apoptosis rate when the OGD group was compared with the Control group (28.50±0.50% vs. 6.13±0.90%, *p*<0.001) (Figure 7A). Compared with the OGD group, the OGD+IL-10/R9-12h group and OGD+IL-10/R21-24h group showed a lower apoptosis rate (respectively, 23.57±0.52% vs. 28.50±0.50%, *p*<0.01; 16.67±1.62% vs. 28.50±0.50%, *p*<0.001) (Figure 7A). Compared with either the OGD+IL-10/R9-12h group or the OGD+IL-10/R21-24h group, the OGD+IL-10/R9-24h group reported a markedly lower apoptosis rate (respectively, 11.73±0.52% vs. 23.57±0.52%, *p*<0.01; 11.73±0.52% vs. 16.67±1.62%, *p*<0.05) (Figure 7A).

Then the expression of Bax and Bcl-xL was detected by western blotting to evaluate the apoptosis level of each group. We found that the OGD treatment increased the expression of Bax, but decreased the expression of Bcl-xL when the OGD group was compared with the Control group (Bax, 2.07±0.16 vs. 0.32±0.13, *p*<0.001; Bcl-xL, 0.44±0.10 vs. 1.40±0.06, *p*<0.001) (Figure 7B, 7C, 7D). When compared with the OGD group, both the OGD+IL-10/R9-12h group and the OGD+IL-10/R21-24h group displayed a lower Bax expression and a higher Bcl-xL expression (respectively, for Bax, 1.29±0.11 vs. 2.07±0.16, *p*<0.01; 0.85±0.12 vs. 2.07±0.16, *p*<0.001; for Bcl-xL, 0.87±0.06 vs. 0.44±0.10, *p*<0.001; 1.09±0.03 vs. 0.44±0.10, *p*<0.001) (Figure 7B, 7C, 7D). Compared with the OGD+IL-10/R9-12h group, the OGD+IL-10/R9-24h group reported a lower expression of Bax and a higher expression of Bcl-xL (Bax, 0.42±0.13 vs. 1.29±0.11, *p*<0.01; Bcl-xL, 1.32±0.06 vs. 0.87±0.06, *p*<0.01) (Figure 7B–7D). When compared with the OGD+IL-10/R21-24h group, the OGD+IL-10/R9-24h group also showed a lower expression of Bax and a higher expression of Bcl-xL (Bax, 0.42±0.13 vs. 0.85±0.12, *p*<0.05; Bcl-xL, 1.32±0.06 vs. 1.09±0.03, *p*<0.05) (Figure 7B–7D).

These results indicate that the treatment with IL-10 at 9-24 h after OGD produces the strongest anti-apoptotic effect by inducing a lower expression of Bax and a higher expression of Bcl-xL in the cortical neurons.

## DISCUSSION

In this study, we demonstrated that p65 was rapidly activated and peaked at 3 h after OGD injury while c-Rel was activated at a late stage and peaked at 24 h after OGD injury. The study also found IL-10 increased neuronal apoptosis if it was administrated at 0-3h after OGD but prevented neuronal apoptosis if it was used at 9–12 h or 21–24 h after OGD. Of interest, IL-10 administration at 0–3 h after OGD significantly activated p65 and promoted apoptosis by enhancing Bax expression and reducing Bcl-xL expression in cortical neurons, which were partly abolished by NF-κB inhibitor PDTC but facilitated by NF-κB promoter PMA; IL-10 administration at 21–24 h after OGD, however, activated c-Rel and attenuated the apoptosis by decreasing Bax expression and increasing Bcl-xL expression in neurons, which were also partly abolished by PDTC but facilitated by PMA. Moreover, the IL-10 treatment at 9–24 h after OGD results in the strongest anti-apoptotic effect by inducing a lower expression of Bax and a higher expression of Bcl-xL in cortical neurons.

Ischemic brain damage has been documented to induce 2 peaks of NF-κB activity in the brain after ischemia [12]. Other studies report that 2 hours after MCAO, activated p65 is mainly found in neurons [22, 23]. Another study also demonstrates that 3 hours after OGD, p65 is highly activated but c-Rel is not changed in neurons [21]. Adding to the existing findings, the current study provides direct evidence to support that in the cultured cortical neurons, p65 subunit, but not c-Rel subunit, is activated immediately after OGD injury, which peaked at 3 h after OGD while c-Rel subunit is significantly activated at a late stage, rather than an early stage, after OGD injury, which peaked at 24 h after OGD. These results show that p65 and c-Rel can be activated and peak at different stages in OGD-injured neurons.

As a traditional protective factor in cerebral ischemia, IL-10 has been found to suppress local inflammatory reactions and decrease neuronal damages in vivo and in vitro [4–6, 8, 9]. After the ischemic stroke, it can reduce the infarct volume by 40% in the IL-10 transgenic mice when compared with that of the wild type mice [5]. Our previous studies also show that IL-10 promotes neuronal survival and neurite outgrowth after OGD via the JAK1/STAT3 and PI3K/Akt pathway [8, 9]. However, different from the traditional view, the current study found that IL-10 increased neuronal apoptosis if administrated at 0-3 h after OGD but reduced the neuronal apoptosis if administered at 9-12 h or 21–24 h after OGD. These results indicate that IL-10 has a dual effect on neuronal survival after OGD injury.

Why does IL-10 play a dual role in neuronal survival after an OGD-induced injury? Available evidence reveals that IL-10 can induce NF-κB activation in neurons [10, 11, 24] and hypoxia-ischemia can induce two peaks of NF-κB activity in the brain: an early NF-κB activation contributing to neuronal damage and a late activation resulting in protection for neurons [12]. We speculated that the dual effect of IL-10 is achieved through the mediation of NF-κB activation. Among the subunits of NF-κB, p65 and c-Rel can be a functional ‘‘end point’’ in cerebral ischemia, dictating neuron death or survival in response to external stimuli [25]. p65 activity contributes to ischemic neuronal damage, while c-Rel promotes neuronal resistance to hypoxia [21]. In the current study, we demonstrated that p65 was mainly activated at an early OGD phase and c-Rel at a late phase. According to the facts above, we further speculated that p65 and c-Rel were involved in the observed dual effect of IL-10 on neuronal survival at different OGD stages. To verify this speculation, we conducted experiments at an early stage and at a late stage, respectively.

At the early OGD stage, we found that IL-10 increased the expression and nuclear translocation of p65 in OGD-injured neurons. Moreover, it promoted OGD-induced apoptosis in neurons by up-regulating the expression of pro-apoptotic protein Bax and down-regulating the anti-apoptotic protein Bcl-xL expression. Of note, NF-κB inhibitor PDTC decreased the neuronal apoptosis by preventing IL-10-increased Bax expression and IL-10-reduced Bcl-xL expression. NF-κB activator PMA increased the neuron apoptosis by promoting IL-10-increased Bax and IL-10-reduced Bcl-xL expression. These results indicate that p65 is involved in the pro-apoptotic effect of IL-10 at an early OGD stage, which is in line with the previous findings. The existing literature documents that neurotoxic stimuli, such as ischemia [21, 23], glutamate [26], β-amyloid [27, 28], or 1-methyl-4-phenylpyridinium (MPP^+^) [29, 30], can induce p65 activation and the transcription of a panel of pro-apoptotic genes in neurons. In cerebral ischemia, p65 activity is associated with an imbalanced expression of pro-apoptotic target genes, such as an increased expression of Bim, Noxa and Bax genes [21, 23]. Bax, as a pro-apoptotic protein, plays an important role in the apoptosis of neurons during the cerebral ischemia [31] and Bcl-xL, as an anti-apoptotic protein, is crucial for decreasing neuronal apoptosis in cerebral ischemia [32], which are further confirmed in the current study. Taken together, the results from the current study reveal that IL-10 remarkably enhances the apoptosis of neurons at an early OGD stage by activating p65, which in turn up-regulates Bax expression and down-regulates Bcl-xL expression and that the inhibition of the early p65 activity by PDTC reduces the IL-10-induced neuronal damage after OGD injury.

Previous research shows that diverse neurotoxic settings, such as S100B in models of NMDA-mediated excitotoxicity [33], agonists at mGlu5 receptors against β-amyloid [27] and MPP ^+^ toxicity [29] or IL-1β against glutamate-mediated cell death [20], can activate the c-Rel expression, which in turn up-regulates the expression of anti-apoptotic genes, facilitating the neuroprotection. Moreover, further evidence shows that the inhibition of c-Rel can decrease Bcl-xL expression against hypoxia-induced cell death in the hippocampus [34]. Also, in cortical neurons, Leptin exerts neuroprotection through c-Rel–dependent transcription of Bcl-xL against OGD-induced apoptosis [35]. Nevertheless, little is known about the effect of c-Rel activation on Bax expression. In this study, we found that IL-10 increased c-Rel expression and c-Rel nuclear translocation in OGD-injured neurons at a late OGD stage. Moreover, IL-10 reduced OGD-induced apoptosis in neurons by down-regulating the expression of pro-apoptotic protein Bax and up-regulating the anti-apoptotic protein Bcl-xL expression. Besides, PDTC increased neuronal apoptosis by preventing IL-10-decreased Bax expression and IL-10-increaced Bcl-xL expression, and PMA decreased neuron apoptosis by promoting IL-10-decreased Bax expression and IL-10-increaced Bcl-xL expression. Taken together, these findings demonstrate that IL-10 remarkably reduces neuronal apoptosis at a late OGD stage by activating c-Rel, which in turn up-regulates Bcl-xL expression and down-regulates Bax expression and that the activation of a late c-Rel activity by PMA facilitates the IL-10-conferred neuroprotection of OGD-injured neurons.

We have found that IL-10 promotes neuronal survival at 9–12 h and 21–-24 h after OGD injury. We wondered whether IL-10 administration at 9–24 h induced a stronger neuronal protection and found that the apoptotic rate at 9–24h markedly declined when compared with that at 9–12 h and 21–24 h. The IL-10/R9-24h group also showed a lower expression of Bax and a higher expression of Bcl-xL than the IL-10/R9-12h or IL-10/R21-24h group. Taken together, the current study demonstrates that for OGD-injured neurons, the administration of IL-10 at a middle-to-late OGD stage can produce the strongest neuronal protective effect. Therefore, IL-10 treatment should be withheld until a middle-to-late stage after ischemic insult in order to preserve protective NF-κB–dependent mechanisms that occur in a later ischemic phase. Clinical applications of IL-10 should be restricted to cases of well-documented acute cerebral infarction, in which detailed records of timing of the insult are available. Our study suggests that with proper timing, IL-10 treatment may be a very effective strategy to combat brain damage after stroke.

On the basis of the above analysis and discussion, the current study concludes that IL-10 treatment can exert an early pro-apoptotic and a late anti-apoptotic effect on the OGD-injured neurons respectively by activating p65 subunit at an early OGD phase and c-Rel subunit at a mid-to-late OGD stage. The optimal neuroprotection appears when IL-10 is administered at 9-24 h after OGD injury. Different from the traditional view of the neuroprotective effect of IL-10, these findings broaden our understanding of the role of IL-10 in cerebral ischemia and provide empirical evidence for its therapeutic value. In treating cerebral ischemia, an early administration of IL-10 can produce adverse effect and a later application in combination with NF-κB activator PMA is recommended. Meanwhile, there are some limitations in our study: we only testified the dual effect of IL-10 on neuronal survival in vitro. Future studies should focus on its effects in vivo. Nevertheless, the study extends the understanding of the neuroprotective effect of IL-10 and suggests its potential therapeutic strategies against cerebral ischemia.

## MATERIALS AND METHODS

### Animals

Pregnant Sprague Dawley rats were provided by the Animal Center of Fujian Medical University (Fuzhou, China), housed in a controlled environment (23 ±1°C and 50% ± 5% humidity) and allowed food and water ad libitum. The room was lighted between 8:00 a.m. and 8:00 p.m. All experimental animals were euthanized with isoflurane, which contained 3% induction and 1.5% maintenance in 30% O_2_ and 70% N_2_O. Experimental protocols followed the *Guidelines of the National Institute of Health* (NIH Publications No. 80-23, revised in 1996) and were approved by Institutional Animal Care and Use Committee of Fujian Medical University.

### Primary cortical neuron cultures

Primary cortical neurons were cultured as described previously [36, 37]. Samples of cerebral cortex from brains of Sprague-Dawley rat embryos (aged 16–18 days) were dissected and trypsinized cells were cultured in a neurobasal medium (Gibco, USA) with 2% B27 (Gibco, USA) supplement, 0.5mM glutamine, 50units/ml penicillin. After 24 hours of incubation in a chamber at 37 °C with 5% CO_2_, the neurobasal medium was refreshed. The purity of the neuronal cultures was confirmed by class III-β -Tubulin and Hoechst 33342 staining, which indicated about 90% of cultured neurons.

### Oxygen-glucose deprivation/reoxygenation

The oxygen-glucose deprivation (OGD) model was established as described previously with minor modifications [38]. Cells were cultured for seven days before exposure to OGD. In brief, the neurobasal medium was replaced with a glucose-free DMEM (Gibco, USA) and then maintained in an incubator containing 5% CO_2_ and 95% N_2_ at 37°C to induce oxygen deprivation. Two hours later, OGD was terminated by replacing the glucose-free DMEM with the original medium and then cells were incubated in their original culture condition for reoxygenation. Control cells were not exposed to OGD conditions.

### Drug treatment

IL-10 from rat recombinant was purchased from PeproTech. It was administered to the cultured cortical neurons after OGD, reaching a final concentration of 20 ng/ml. For neurons treated with IL-10, PDTC (Abcam, UK), a specific inhibitor of NF-κB [39, 40], or PMA (Abcam, UK), an activator of NF-κB [41, 42], was simultaneously administered, respectively reaching a final concentration of 20uM and of 20ng/ml. The OGD group was vehicle-treated in the right time after OGD.

To evaluate the effect of IL-10 on the apoptosis of cortical neurons at different stages after OGD injury, we treated neurons with IL-10 at 0–3 h, 3–6 h, 9–12 h, 21–24 h and 33–36 h after OGD, respectively. The experimental groups were designed as follows: Control group, OGD group, and OGD+IL-10/R0-3h group, OGD+IL-10/R3-6h group, OGD+IL-10/R9-12h group, OGD+IL-10/R21-24h group, OGD+IL-10/R33-36h.

In our preliminary experiments, the administration of IL-10 induced the greatest pro-apoptotic effect at 0–3 h after OGD and the best anti-apoptotic effect at 21–24 h after OGD (Figure 2). Therefore, 0-3 h and 21–24 h were respectively indicated as the early stage and late stage of OGD injury. To further determine the mechanism underlying the early pro-apoptosis and late anti-apoptosis of IL-10, PDTC or PMA was simultaneously administered, along with IL-10, to the cultured cortical neurons at 0–3 h and 21–24 h after OGD. The culture medium was respectively changed to a neuronal medium which contained no IL-10, PDTC, and PMA after 3 hours of drug treatment. The experimental groups were as follows: for the early stage, Control group, OGD group, OGD+IL-10/R0-3h group, OGD+IL-10/R0-3h+PDTC/R0-3h group, and OGD+IL-10/R0-3h+PMA/R0-3h group; for the late stage, Control group, OGD group, OGD+IL-10/R21-24h group, OGD+IL-10/R21-24h+PDTC/R21-24h group, OGD+IL-10/R21-24h+PMA/R21-24h group.

### Flow cytometry using Annexin V/PI staining

To detect the apoptosis of neurons quantitatively, we performed flow cytometry as described previously with modifications [43]. Fluorescein Annexin V-FITC/PI double labeling was performed with an Annexin V-FITC apoptosis detection kit (Beyotime, China). Briefly, neurons were seeded in culture flasks (25cm²). Forty-eight hours after OGD, the cells were stained with Annexin V-FITC and PI according to the instruction of the manufacturer. The apoptotic cells were determined with a flow cytometer (Beckton Dickinson, USA). The nuclear translocation of p65 and c-Rel was quantified using the Fiji/Image J [44, 45].

### Western blot analysis

Western blot analysis was performed as described to analyze the expression of p65, c-Rel, Bcl-xL, Bax and β-Actin in neurons from each group [46]. Cell extracts were collected on ice in a RIPA lysis buffer (Beyotime, China) and then centrifuged at 14000×g at 4°C for 10 min. Proteins were separated on 12% SDS-polyacrylamide gels and then electrotransferred onto a PVDF membrane (Millipore, USA). The membranes were blocked in 5% non-fat milk in TBS and probed with primary antibodies to rabbit anti-p65 (1:500, Santa Cruz, USA), rabbit anti-c-Rel (1:500, Santa Cruz, USA), rabbit anti-Bcl-xL (1:1000, Abcam, UK), rabbit anti-Bax (1:1000, Abcam, UK), rabbit anti-β-Actin (1:1000, Abcam, UK) and then incubated with a goat anti-rabbit IgG-HRP secondary antibody (1:8000, Abcam, UK). The optical densities of the antibody-specific bands were detected with ECL reagent kits and analyzed with Image J software (1.46r).

### Assessment of cell survival by MTT extraction assay

Cell survival was quantified by the tetrazoliumsalt-extraction method as previously described [47]. Because viable cells with active mitochondria can cleave the tetrazolium ring into a visible dark-blue formazan reaction product, 3,(4,5-dimethyldiazol-2-yl)2,5-diphenyl-tetrazolium bromide (MTT) was added to the culture medium at a final concentration of 5 mg/ml and incubated at 37°C for 4 hours and OD was measured at 570 nm using a microplate reader (Multiskan FC, Thermo, USA).

### Immunofluorescence staining

Immunofluorescence staining was performed to locate the expression p65 and c-Rel as described previously [48, 49]. It was applied to cells 3 hours after the drug treatments as described**.** Cells were washed in PBS at 37°C and fixed on 4% paraformaldehyde. Then corresponding antibodies were used to incubate neurons at 4°C overnight. After three washes with PBS, the neurons were further incubated with secondary antibodies at room temperature for 2 hours. Nuclei were stained by hoechst33342 (5 μ g/ml, Sigma, USA) and visualized under a ZEISS LSM 780 (Carl Zeiss, Germany).

### Statistical analysis

All data were presented as mean±SEM and analyzed with SPSS17.0 statistical software (IBM, USA). Each measurement was performed in replication of 3 independent trials. One-way analysis of variance (ANOVA) was performed for statistical significance among groups; Student-Newman-Keuls multiple comparison test was conducted when equal variances were assumed and Dunnett’s T3 was applied when equal variances was not assumed; unpaired two-tailed Student’s t-tests were employed to determine the significance of mean differences between two groups. *P* values less than 0.05 (two-sided) were considered as statistically significant.
